# The UCSC Genome Browser database: 2018 update

**DOI:** 10.1093/nar/gkx1020

**Published:** 2017-11-02

**Authors:** Jonathan Casper, Ann S Zweig, Chris Villarreal, Cath Tyner, Matthew L Speir, Kate R Rosenbloom, Brian J Raney, Christopher M Lee, Brian T Lee, Donna Karolchik, Angie S Hinrichs, Maximilian Haeussler, Luvina Guruvadoo, Jairo Navarro Gonzalez, David Gibson, Ian T Fiddes, Christopher Eisenhart, Mark Diekhans, Hiram Clawson, Galt P Barber, Joel Armstrong, David Haussler, Robert M Kuhn, W James Kent

**Affiliations:** 1 Genomics Institute, University of California Santa Cruz, Santa Cruz, CA 95064, USA; 2 Howard Hughes Medical Institute, University of California Santa Cruz, Santa Cruz, CA 95064, USA

## Abstract

The UCSC Genome Browser (https://genome.ucsc.edu) provides a web interface for exploring annotated genome assemblies. The assemblies and annotation tracks are updated on an ongoing basis—12 assemblies and more than 28 tracks were added in the past year. Two recent additions are a display of CRISPR/Cas9 guide sequences and an interactive navigator for gene interactions. Other upgrades from the past year include a command-line version of the Variant Annotation Integrator, support for Human Genome Variation Society variant nomenclature input and output, and a revised highlighting tool that now supports multiple simultaneous regions and colors.

## INTRODUCTION

The UCSC Genome Browser (1) was first released in 2001 as a tool to display the then newly assembled human genome. It has grown since then to accommodate new assemblies and forms of annotation, and it now provides browsers for more than 180 assemblies and over 100 species. The Browser’s team provides training workshops worldwide to help its users learn to take advantage of the latest features.

Updates to the Browser fall into two primary categories: data and tools. Data updates include new assemblies and new tracks on existing assemblies, but also track hubs from external data providers that are added to UCSC’s displayed list of public track hubs. Tool updates are improvements that allow the display of new types of data or permit users to perform new types of analysis.

## NEW DATA AND DISPLAYS

In the past year, 12 new assemblies were added along with more than 28 track updates to existing assemblies (see Supplementary Tables S1 and S2 for details). A few of these track additions deserve special mention, like the CRISPR/Cas9 binding site track, the new gene interactions display, and the update to the RefSeq Genes track to display NCBI’s mappings alongside UCSC’s own alignments. The track configuration page for the existing Genotype-Tissue Expression (GTEx) project tracks have been upgraded, and now feature an interactive body map. Finally, the track hub portal has received a number of additions in the past year, including a UCSC-created hub containing 16 mouse strain assemblies.

### CRISPR/Cas9 track release

**Figure 1.**
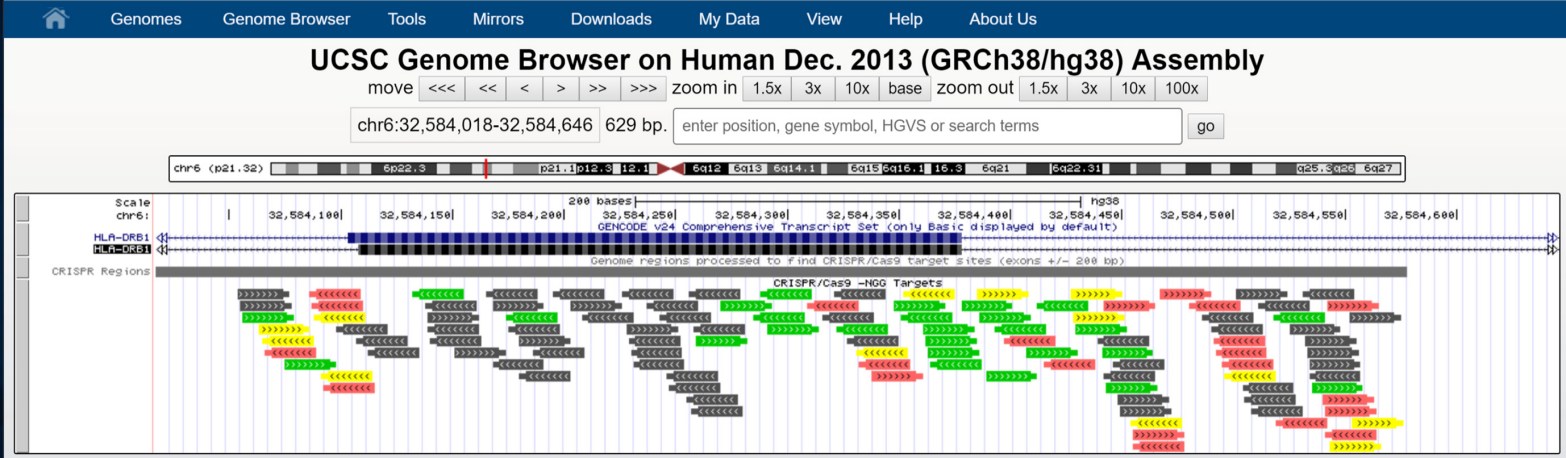
CRISPR/Cas9 Track. This track displays binding sites for the CRISPR/Cas9 complex that fall in transcribed regions or within 200 bp of such regions. Sites are scored for predicted cleavage efficiency and specificity and colored accordingly. Green sites have high predicted cleavage efficiency and specificity. Gray sites have low specificity.

One of the latest additions to the Browser is the CRISPR/Cas9 track on human and model organism assemblies. CRISPR is a genome editing technique adapted from bacterial immune systems. The technique depends on finding guide sequences of RNA that successfully bind to the region to be edited while avoiding other genomic regions. The new track is designed to assist in the search for appropriate guide sequences by listing potential binding sites for the CRISPR/Cas9 complex that are near transcribed regions (2). Specifically, sites are listed in the track if they fall within a transcribed region or are within 200 bp of one. For each site, the track provides possible guide sequences along with a collection of predicted efficiency and specificity scores for those guide sequences. It also provides information about potential off-targets for each guide sequence, grouped by the number of mismatches between the off-target sites and the guide (Figure 1).

Sensitivity and specificity score calculations for each site were provided by tools from CRISPOR (http://crispor.tefor.net). The list of scores provided includes a guide specificity score, an out-of-frame score (3) and efficiency scores (4,5).

The CRISPR/Cas9 track has been made available for the following assemblies: human (hg38, hg19), mouse (mm10, mm9), rat (rn5), zebrafish (danRer7), *Caenorhabditis elegans* (ce10), *Drosophila melanogaster* (dm6), yeast (sacCer3) and *Ciona intestinalis* (ci2). If there is enough interest from the research community, additional tracks may be created for other assemblies.

### Gene Interactions track release

The Gene Interactions track for the human GRCh38/hg38 and GRCh37/hg19 assemblies presents information about cellular pathways and regulatory networks. Configuration options for this track can be found in the Phenotype and Literature track group. Each item in the track corresponds to a gene and provides a list of other genes that interact with it.

**Figure 2.**
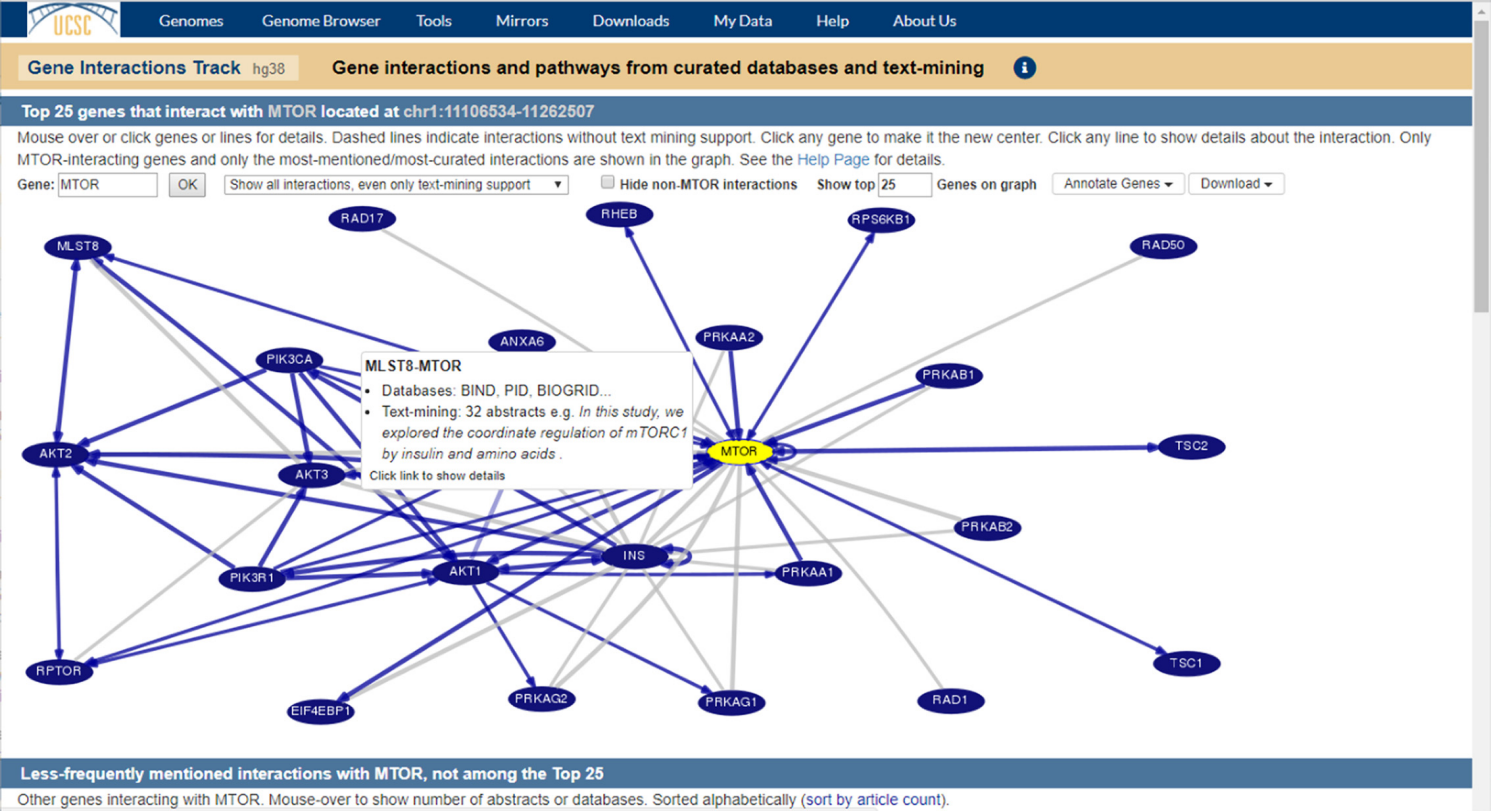
Gene Interactions Tool. This tool displays the network of gene interactions as determined from a collection of 23 pathway and protein-interaction databases, in tandem with text-mining of abstracts on PubMed. Hovering over the edge between two genes (shown) shows information about the interaction between them. Clicking on an edge provides more details on a separate page.

Clicking on a gene in this track allows a user to explore the interaction data for that gene in a new graph visualization tool. Genes in this tool are represented by nodes in the graph, with the selected gene highlighted in yellow (Figure 2). A user can click any gene in the graph to re-center the interactions tool on that gene. An edge between two nodes in the graph indicates that those two genes interact. Hovering the mouse pointer over an edge displays a pop-up with a summary of the sources that report that gene interaction, while clicking on the edge takes the user to a more detailed description page with links to the databases and PubMed abstracts of publications that support the interaction.

The data for this track were pulled from 23 curated databases, including iRefIndex 13 (6–20), String 9.1 (21,22), WikiPathways (23,24), the OpenBEL large corpus (https://github.com/OpenBEL/openbel-framework-resources/tree/latest/knowledge) and others (25–35), and were augmented by text mining of PubMed abstracts. The full list of databases examined is provided in Supplementary Table S3. The text-mined abstract data were provided by Chris Quirk and Hoifung Poon as part of Microsoft Research’s Project Hanover (36).

### RefSeq Genes track update: now with NCBI mappings

The RefSeq Genes track at UCSC displays gene transcripts from the NCBI Reference Sequence Database (37). Historically, this track has displayed UCSC’s BLAT (38) alignments of transcript sequences to the genome assembly instead of the mappings provided by RefSeq. This sometimes led to confusion when UCSC’s alignments diverged from those provided by NCBI (i.e. in areas that are difficult to align). As an added issue, the previous version of the track only mapped RefSeq records with the NM_ and NR_ accession prefixes (denoting RNA/mRNA transcripts that have been experimentally verified). Unfortunately, this completely excluded mitochondrial annotation.

To resolve these issues, the RefSeq genes track has been redesigned for the GRCh38/hg38 human genome assembly. It now contains a collection of subtracks that display NCBI’s mappings for the various accession classes: curated, predicted and otherwise. A companion subtrack is available for those users who wish to continue to use UCSC’s mappings.

### GTEx Gene Track update: body map selection tool

**Figure 3.**
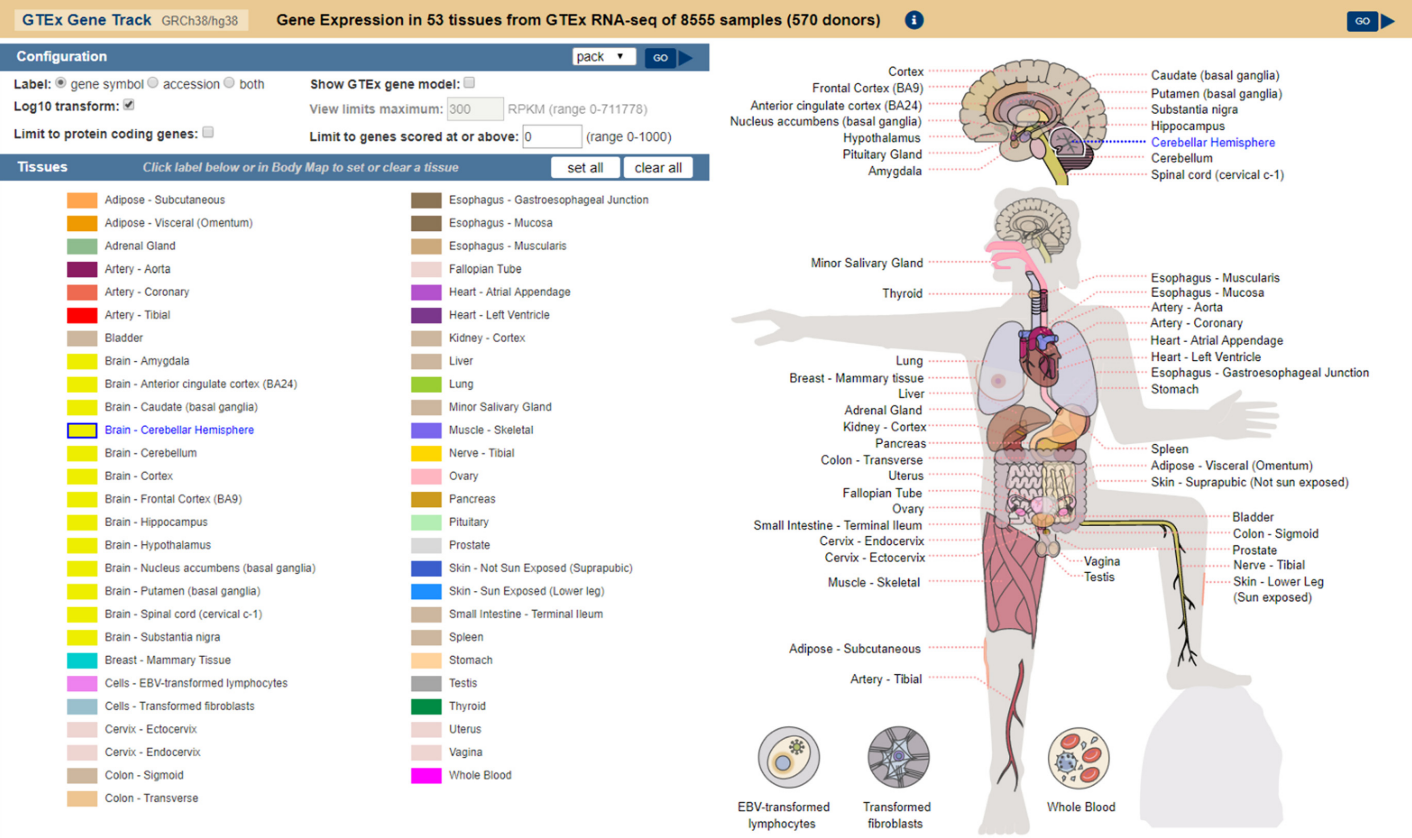
New Genotype-Tissues Expression (GTEx) track interface. The previous checklist interface remains on the left. The body map on the right is interactive; hovering the mouse pointer over a tissue name will highlight that tissue in the body map image. Tissue names can be clicked to activate or deactivate that tissue in the track display, just as with the checklist.

The GTEx Gene track on human genome assemblies displays expression levels of genes in different tissue types as determined by the NIH Genotype-Tissue Expression project (https://commonfund.nih.gov/GTEx). Previously, the configuration page for this track was just a plain checklist of tissue types, which could make it difficult to locate tissues of interest. A revised interface now makes tissue selection much easier by providing a visual body map with tissue labels grouped near the corresponding region of the body (Figure 3). A checkbox selection menu remains available for users who prefer that interface.

### Track hub portal updates

The Genome Browser team maintains a list of public track hubs (39) for the convenience of the community. Users are welcome to submit their own hubs for inclusion by contacting the Browser team. Last year seven new hubs were added to this public listing, including three assembly hubs with genomes that are not natively displayed on the Browser. For example, one of the new hubs is a Mouse Strains hub constructed by UCSC using data from the Mouse Genomes Project (http://www.sanger.ac.uk/science/data/mouse-genomes-project). The hub provides browsers for sixteen different strains of mice, each as a separate assembly with its own annotation that includes genes, repeat sequences and other basic tracks. The hub also provides a multiple alignment track on the native GRCm38/mm10 mouse genome assembly, showing the alignment of all 16 strains against each other and the mm10 assembly. A full list of hubs added in the last year is available in Supplementary Table S4.

## TOOL IMPROVEMENTS

Software updates to the UCSC Genome Browser in previous years have included tools like the Genome Browser in a Box (GBiB) (40) and the Variant Annotation Integrator (VAI) (41). Those tools have been expanded in the last year to support greater numbers of users. GBiB can now access data from our European mirror server to improve performance, and there is now a command-line version of the VAI for working with data files locally.

There have also been several upgrades to the Browser interface. There is a new track type: barChart, which mimics the display of the bar chart graphs seen in the GTEx tracks. The position box now supports a subset of the Human Genome Variation Society (HGVS) sequence variant nomenclature as well as similar notations. Hub support has been improved, with new options for building hubs and greater detail about matches in hub search results. The highlighting tool for indicating regions of interest in a Browser image now permits any number of regions to be highlighted instead of just one, and each highlight can be given a different color from the RGB color spectrum. Finally, there is now a way to store BLAT sequence alignment results past the previous 48 h expiration policy.

### European public MySQL server with GBiB support

The European mirror (https://genome-euro.ucsc.edu) of the UCSC Genome Browser was upgraded this year, and now provides a public MySQL server in addition to the interactive web-based Browser. The server can be found at genome-euro-mysql.soe.ucsc.edu, with the same credentials and access restrictions that apply to the US-based MySQL server (for details, see http://genome.ucsc.edu/goldenPath/help/mysql.html).

GBiB is now configured to take advantage of this MySQL server as well. Previously, GBiB was restricted to fetching data from UCSC’s servers on the west coast of the United States. For users in other countries this could result in unacceptable delays unless they first chose to download entire tracks of data from UCSC—a time-consuming (and space-consuming!) proposition. With the new European MySQL server, GBiB now has two options for interactively fetching the data that it needs. GBiB users can switch seamlessly between the two servers depending on which gives the best response time. UCSC has also replicated a small selection of files from its download server (the ‘gbdb’ directory) for faster GBiB access. Together, this should substantially improve performance for GBiB users in European countries.

### Command-line version of the Variant Annotation Integrator

Four years ago UCSC released the web-based Variant Annotation Integrator (VAI): a tool for taking a list of genomic variants and predicting their functional consequences. Being web-based means the VAI fits nicely into the Browser’s suite of online analysis tools, but also means it has certain limitations. For example, users may be unwilling or unable to upload their variant data to UCSC’s servers for analysis. To address this shortcoming, there is now a command-line version of the VAI called *vai.pl*.

The *vai.pl* tool can be run either from the virtual machine GBiB or from a mirror of the UCSC Genome Browser. This means that variant data never leaves the user’s machine when using *vai.pl*. The 100k variant limit imposed by the web version of the tool can also by bypassed for the command-line version, allowing analysis of a substantially larger number of variants. More information on how to use *vai.pl* can be found in the Browser team’s blog post at http://genome.ucsc.edu/blog/annotating-millions-of-private-variants-with-vai-pl/. *vai.pl* is available as part the GBiB and GBiC (see below) products and is also included in the UCSC Genome Browser source code (https://genome-store.ucsc.edu).

### GBiC: Genome Browser in the cloud

The Genome Browser in a Box tool provides a packaged virtual machine version of the UCSC Genome Browser that can be run on a user’s computer. There is now a related package available titled GBiC: Genome Browser in the Cloud. GBiC is an installation script that transforms a bare Debian-based or RedHat-based Linux server into a mirror of the UCSC Genome Browser, including handling the installation of MySQL and Apache. The script is suitable for being run on fresh virtual machines in a cloud system such as Amazon AWS (https://aws.amazon.com/) or Microsoft Azure (https://azure.microsoft.com/), so that a new, fully functioning mirror of the Browser can be set up in less than an hour. The script also handles data mirroring and updating of the Browser software for these virtual machines.

### New track type: barChart

The Genotype-Tissue Expression tracks on human assemblies involved a new form of display for the browser: bar charts within the Browser display (42). This display format is now available to all users for building custom tracks and track hubs. In addition to providing a bar chart display within the Browser, this track format also allows users to create boxplots on details pages. While the bar chart is suitable for a quick display of median values from expression data, the box plots provide a more complete view of the data distributions. More information about using this track format can be found at http://genome.ucsc.edu/goldenPath/help/barChart.html.

### Human Genome Variation Society (HGVS) term support

Variant descriptions using the HGVS variant nomenclature (43) are now recognized by the UCSC Genome Browser. For example, from the Browser gateway page at http://genome.ucsc.edu/cgi-bin/hgGateway, selecting the human GRCh38/hg38 genome assembly and entering NM_005915.5:c.1917+326C>T into the search box will locate and highlight a variant in the MCM6 gene. The Browser automatically highlights the position of variants identified by HGVS terms. Currently recognized HGVS variant classes include ‘p.’ (protein), ‘g.’ (genomic), ‘c.’ (coding), and ‘n.’ (non-coding).

In a complementary vein, an update to the Variant Annotation Integrator allows it to generate HGVS terms for a user’s list of variants. Options exist for generating all of the same term types that are currently recognized by the Browser. A command-line version of this function is also available in the tool *vcfToHgvs*, which can be found with the rest of UCSC’s utilities in the Genome Browser in a Box and source code package releases (https://genome-store.ucsc.edu).

### Track hub portal search improvements

**Figure 4.**
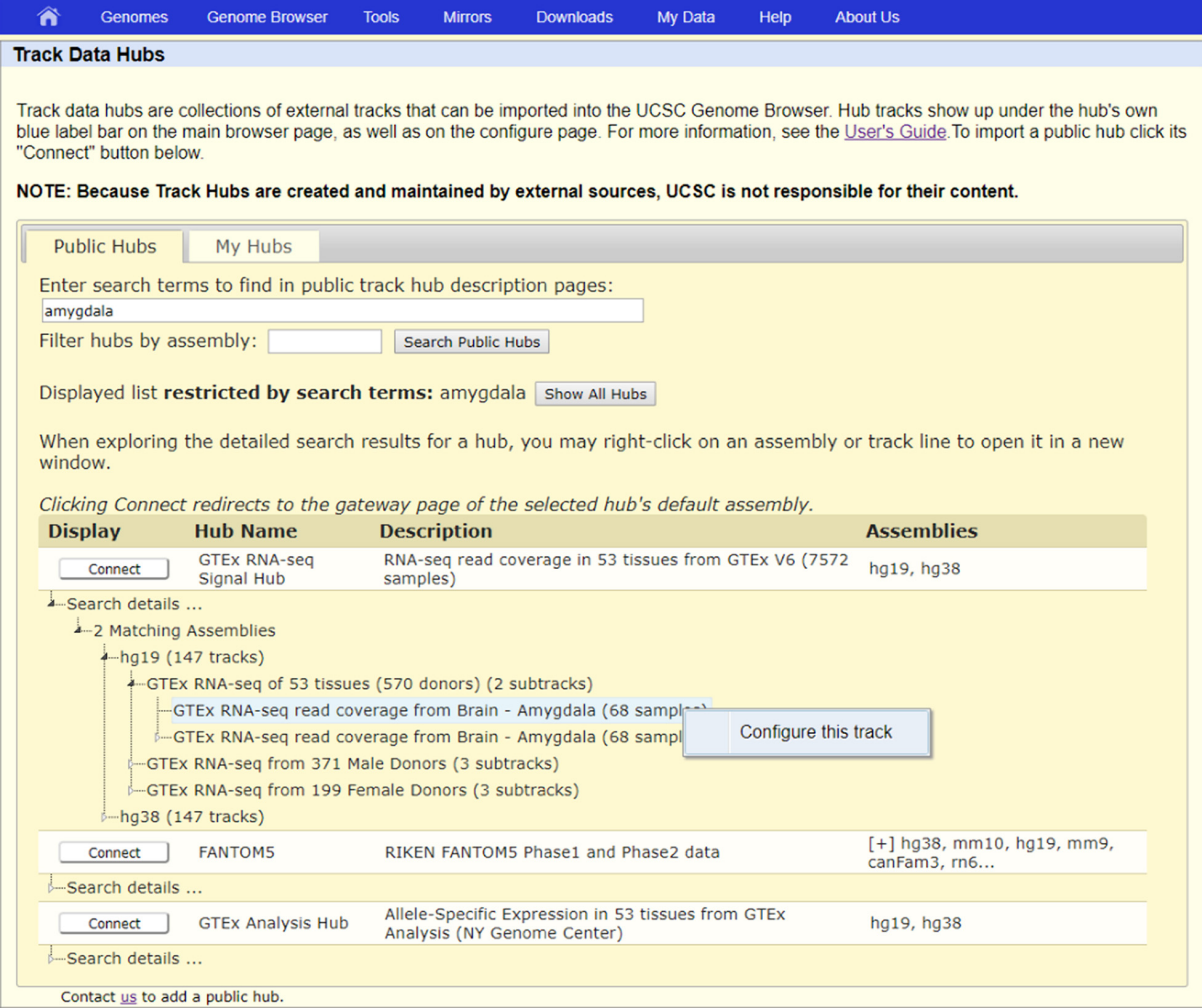
Example hub search results. This shows an example track hierarchy in the search results for the GTEx RNA-seq hub, looking for tracks that match the term ‘amygdala’. The highlighted track has been right-clicked, and clicking the ‘Configure this track’ link will connect to the hub and open the configuration page for that track.

The hub portal now has a significantly improved search tool (Figure 4). Previously, a search on the public hubs listing would return a terse list of hubs that matched the search terms. That list has been expanded to list all matching assemblies and tracks within each hub, arranged hierarchically. All assemblies and tracks in the search results can be right-clicked for a link to connect directly to the hub and immediately begin variously viewing the assembly or configuring the track. In addition, the body of text searched for each hub has been expanded to include the text of the description pages for hubs, their assemblies (for assembly hubs) and their tracks.

### Track hub backend upgrades

The Browser is now able to connect to track hub data on Amazon AWS HIPAA compliant storage (https://aws.amazon.com/compliance/hipaa-compliance/) and other similar cloud storage solutions. This provides more options for users who would like to use GBiB/GBiC or a local Browser mirror to analyze protected data without storing it on the same server as the GBiB/GBiC or mirror installation. It is important to note that while UCSC’s public Browser sites can also access data from these systems, UCSC does not provide any guarantee of data security on its public servers.

New track types have been added in version 2 of the official Track Hub Specification (https://genome.ucsc.edu/goldenPath/help/trackDb/trackDbHub.html). The additions include bigBarChart, bigMaf, bigChain, bigPsl, bigGenePred and CRAM (http://www.ebi.ac.uk/ena/software/cram-toolkit). The update also shifts some existing track tags into the ‘base’ support category, indicating that they are likely to be supported by other genome browsers such as Ensembl (44), Biodalliance (45) and the WashU Epigenome Browser (46). The track tags that have been shifted to the ‘base’ support level are *html, priority, colorByStrand, autoScale* and *spectrum*.

### Multi-feature and multi-color highlighting

**Figure 5.**
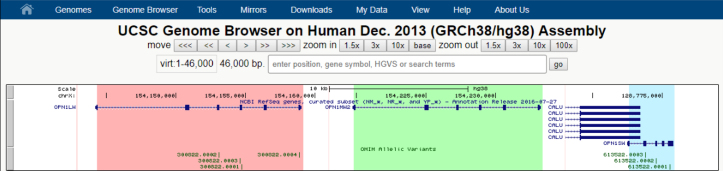
Example of highlighting multiple features with different colors. This image shows the OPN1LW, OPN1MW and OPN1SW genes from chrX and chr7 brought together using the Browser's multi-region mode, each with its own highlight.

In 2014 the Browser released a highlighting tool, allowing users to pick a region of their view and apply a special vertical highlight to it. This was useful for calling out particular features in screenshots of the Browser, but it was limited to highlighting only a single region in one color: light blue. The Browser now supports highlighting multiple regions in any color (Figure 5). To use the tool, click and drag near the top of the Browser image to select a region or hold down the shift key and drag anywhere within the image. A menu will pop up offering options to zoom to the selected region or to highlight it with a variety of colors. The ‘Single highlight’ button removes all other active highlights before creating a new one, while the ‘Add highlight’ button adds the new highlight to the ones that are already present. Highlights can be cleared individually by right-clicking on them and selecting ‘Remove highlight’ from the context menu, or by selecting ‘Remove all highlights’ from the View menu at the top of the page.

### Updated policy for storing BLAT search results

Results from the BLAT sequence alignment tool on the UCSC Genome Browser have historically been stored for only 48 hours after the search was performed. After that period, the search results were deleted even if they were part of a saved session. An update to the BLAT alignment tool now includes a button that offers to transform the BLAT alignment results into a custom track. This custom track will display the results in exactly the same format as standard BLAT results, but will not be subject to the 48 h expiration time. Instead, it will be treated like any other custom track on the Browser. If it is saved as part of a Browser session, the BLAT result custom track will persist as long as the saved session exists. The custom track can also be downloaded for archival purposes using the UCSC Table Browser.

## OUTREACH AND CONTACT INFORMATION

In the last year, the Genome Browser’s training team provided 19 seminars to help its users learn to take advantage of the latest features. Outreach is also supported by regular updates to the training documentation (https://genome.ucsc.edu/training/) and blog (http://genome.ucsc.edu/blog/) with videos and in-depth descriptions of new Browser features. The training documentation also includes information on how to submit a seminar request.

General contact information for the UCSC Genome Browser can be found on the website at https://genome.ucsc.edu/contacts.html, including information for accessing our email support list and the archive of previously answered mailing list questions. UCSC also maintains mirrors in Germany and Japan with the gracious assistance of the University of Bielefeld, Germany and the RIKEN Institute of Japan, respectively. Those sites can be found at https://genome-euro.ucsc.edu, and https://genome-asia.ucsc.edu.

## PLANS FOR THE FUTURE

The UCSC Genome Browser team has several features in mind for the coming year. The immediate focus will be on improving support for assembly patches and haplotype regions. This is a natural extension of the multi-region support that was released in recent years. The goal is to develop mechanisms for mapping annotations from the reference assembly to the corresponding patch and haplotype regions, so that those regions can be displayed *in situ* within the larger assembly.

A larger focus on track hub development is also part of UCSC’s plans. There are two external resources in particular that UCSC intends to work closely with in the coming years: GTEx (47) and ENCODE (http://www.encodeproject.org). Each of these resources already provides a vast amount of data in track hub format, with still more data to be added. As part of this work, the hub specification will be updated to include options for metadata and search tools to locate items within tracks.

## Supplementary Material

gkx1020_supp
