# Supplementary material for: The UCSC Genome Browser database: 2018 update
Source: Nucleic Acids Res. 2017 Nov 2;46(Database issue):D762–9. doi: 10.1093/nar/gkx1020 (PMC5753355; doi:10.1093/nar/gkx1020)
Supplement: gkx1020_supp [file gkx1020_supp.pdf]

Supplemental Data

KEY: N = new, NU = new & updated, U = updated, AU = automatically updated

**Track(s)**	**New/Update Status**	**Human assemblies**	**Mouse assemblies**	**Other assemblies**
GENCODE v26 & v27	N	hg38		
GRCh38 Patch 9 & 11	N	hg38		
GTEx Transcript Expression	N	hg38, hg19		
dbSNP v147 & v150	N	hg38, hg19		
Gene Interactions from Curated Databases and Text-Mining	N	hg38, hg19		
NCBI RefSeq Genes	N	hg38	mm10	
CRISPR/Cas9 - NGG Targets	N	hg38, hg19	mm10	ce10, ci2, criGri1, danRer7, dm6, rn5, sacCer3
TransMap Alignments v4	N	hg38, hg19	mm10	> 60 others
GENCODE VM11 & VM14	N		mm10	
Alternate mouse strain sequences	N		mm10	
20 Species Conservation	N			rn6
Chains & Nets (*for many assemblies*)	N	various	various	various
dbSNP v148	N			bosTau8
Pfam Domains in RefSeq Genes	N			dm6

dbSNP v147	N			galGal5
Ensembl Genes v86 & v89	N			many
Locus Reference Genomic (LRG) Sequences	NU	new for hg38, updated for hg19		
Database of Genomic Variants (DGV): Structural Variation	U	hg38, hg19, hg18		
Pfam Domains in UCSC genes	U	hg19	mm10	
COSMIC v81 & v82	U	hg38, hg19		
GenBank and RefSeq Updates (RefSeq Genes, ESTs, RNAs)	AU	Most assemblies updated	Most assemblies updated	Most assemblies updated
ClinGen CNVs (formerly ISCA)	AU	hg38, hg19		
ClinVar Variants	AU	hg38, hg19		
GeneReviews	AU	hg38, hg19, hg18		
NHGRI-EBI Catalog of Published GWAS	AU	hg38, hg19, hg18		
OMIM Genes & Phenotypes	AU	hg38, hg19, hg18		
DECIPHER	AU	hg19		
GRC Incident Database	AU	hg38, hg19		

Supplementary Table 1. Annotation tracks added or updated within the last year.

UCSC Assembly Name	NCBI Assembly Accession	Organism	Provider
ci3	GCF_000224145.1	C. Intestinalis	Kyoto Dept. of Zoology KH
rhiRox1	GCA_000769185.1	Golden Snub-Nosed Monkey	Novogene
bisBis1	GCA_000754665.1	Bison Bison Bison	Univ. of Maryland
galVar1	GCF_000696425.1	Malayan Flying Lemur	Washington University
aquChr2	GCA_000766835.1	Golden Eagle	Washington University
manPen1	GCA_000738955.1	Chinese Pangolin	Washington University
nanPar1	GCA_000935625.1	Tibetan Frog	Beijing Genomics Institute
nasLar1	GCA_000772465.1	Proboscis Monkey	Proboscis Monkey Functional Genome Consortium
melGal5	GCF_000146605.2	Turkey	Turkey Genome Consortium
panTro5	GCF_000001515.7	Chimpanzee	Chimpanzee Sequencing and Analysis Consortium
gorGor5	GCA_900006654.1	Gorilla	University of Washington
chlSab2	GCA_000409795.2	Green Monkey	Vervet Genomics Consortium

Supplementary Table 2. List of assemblies added to the UCSC Genome Browser in the last year.

Database	Notes
iReflex 13 (1–15)	
Androgen Responsive Gene Database (16)	No longer available online, but UCSC maintains a copy at http://hgdownload.soe.ucsc.edu/goldenPath/external/geneGraph/.
String 9.1 (17, 18)	
Negatome 2.0 (19)	
Corum Protein Complexes (4, 5)	
Gene Ontology Protein Complexes (20)	
KEGG (21–23)	Version from April 2011, before the switch to a non-commercial license.
NCI Pathway Interaction Database (24)	No longer available online in its original format, but UCSC maintains a copy at http://hgdownload.soe.ucsc.edu/goldenPath/external/geneGraph/.
BioCarta (25)	Not directly available in a machine readable format. UCSC maintains a copy of a version from 2009 that was included in the original NCI-PID at http://hgdownload.soe.ucsc.edu/goldenPath/external/geneGraph/.
Reactome 2014 (26, 27)	
WikiPathways (28, 29)	Version 20170510
OpenBEL large corpus (https://github.com/OpenBEL/openbel-framework-resources/tree/latest/knowledge)	Version 20150611 (commit 5515fcf, Jan 2016). This database is Copyright 2011-2015, Selventa and under a non-commercial license.
FastForward (30)	

Supplementary Table 3. List of database resources used to construct the new Gene Interactions track for the GRCh38/hg38 human genome assembly. The track is also supported by text-mining of paper abstracts drawn from PubMed.

Track Hub Name	Provider	Assemblies	Hub URL
Mouse Strain Assemblies	UCSC	mm10, rn6, 129S1_SvImJ, WSB_EiJ, SPRET_EiJ, AKR_J, DBA_2J, CBA_J, A_J, C57BL_6NJ, CAST_EiJ, PWK_PhJ, LP_J, C3H_HeJ, NZO_HlLtJ, NOD_ShiLtJ, FVB_NJ, BALB_cJ	http://hgdownload.soe.ucsc.edu/hubs/mouseStrains/hub.txt
Cotney Lab Human Craniofacial Epigenomics	Cotney Lab at UConn Health	hg19, mm9	https://cotneylab.cam.uchc.edu/~jcotney/CRANIOFACIAL_HUB/Craniofacial_Data_Hub.txt
GeneHancer Enhancers	GeneCard/MalaCards	hg38	https://genecards.weizmann.ac.il/geneloc/gh_hub/hub.txt
Hippocampal DNA Methylation and Gene Transcription	Kyle Schachtschneider, U of Illinois, Chicago	susScr3	http://public.hpcagrogenomics.wur.nl/ABGC/Track_Hubs/Cognition/hub.txt
EPD Viewer Hub	Eukaryotic Promoter Database	amel5, araTha1, zm3, spo2	http://epd.vital-it.ch/ucsc/epdHubCustomSpecies/epdHubCustomSpecies.txt
Umap and Bismap Mappability	Hoffman Lab at the Princess Margaret Genomics Centre	hg38, hg19, mm10, mm9	https://pmgenomics.ca/hoffmanlab/proj/bismap/trackhub/hub.txt
ChromosomeAssembliesGR	Damas et. al.	colLiv2, galGal4, falPer2	http://sftp.rvc.ac.uk/rvcpaper/birdsHUB/hub.txt

Supplementary Table 4. Track and assembly hubs added to UCSC’s Public Hubs listing in the last year.

References

1. Bader,G.D., Betel,D. and Hogue,C.W.V. (2003) BIND: the Biomolecular Interaction Network Database. *Nucleic Acids Res.*, **31**, 248–250.

2. Alfarano,C., Andrade,C.E., Anthony,K., Bahroos,N., Bajec,M., Bantoft,K., Betel,D., Bobechko,B., Boutilier,K., Burgess,E., *et al.* (2005) The Biomolecular Interaction Network Database and related tools 2005 update. *Nucleic Acids Res.*, **33**, D418-424.

3. Chatr-Aryamontri,A., Oughtred,R., Boucher,L., Rust,J., Chang,C., Kolas,N.K., O'Donnell,L., Oster,S., Theesfeld,C., Sellam,A., *et al.* (2017) The BioGRID interaction database: 2017 update. *Nucleic Acids Res.*, **45**, D369–D379.

4. Ruepp,A., Brauner,B., Dunger-Kaltenbach,I., Frishman,G., Montrone,C., Stransky,M., Waegele,B., Schmidt,T., Doudieu,O.N., Stümpflen,V., *et al.* (2008) CORUM: the comprehensive resource of mammalian protein complexes. *Nucleic Acids Res.*, **36**, D646-650.

5. Ruepp,A., Waegele,B., Lechner,M., Brauner,B., Dunger-Kaltenbach,I., Fobo,G., Frishman,G., Montrone,C. and Mewes,H.-W. (2010) CORUM: the comprehensive resource of mammalian protein complexes--2009. *Nucleic Acids Res.*, **38**, D497-501.

6. Salwinski,L., Miller,C.S., Smith,A.J., Pettit,F.K., Bowie,J.U. and Eisenberg,D. (2004) The Database of Interacting Proteins: 2004 update. *Nucleic Acids Res.*, **32**, D449-451.

7. Keshava Prasad,T.S., Goel,R., Kandasamy,K., Keerthikumar,S., Kumar,S., Mathivanan,S., Telikicherla,D., Raju,R., Shafreen,B., Venugopal,A., *et al.* (2009) Human Protein Reference Database--2009 update. *Nucleic Acids Res.*, **37**, D767-772.

8. Breuer,K., Foroushani,A.K., Laird,M.R., Chen,C., Sribnaia,A., Lo,R., Winsor,G.L., Hancock,R.E.W., Brinkman,F.S.L. and Lynn,D.J. (2013) InnateDB: systems biology of innate immunity and beyond--recent updates and continuing curation. *Nucleic Acids Res.*, **41**, D1228-1233.

9. Orchard,S., Ammari,M., Aranda,B., Breuza,L., Briganti,L., Broackes-Carter,F., Campbell,N.H., Chavali,G., Chen,C., del-Toro,N., *et al.* (2014) The MIntAct project--IntAct as a common curation platform for 11 molecular interaction databases. *Nucleic Acids Res.*, **42**, D358-363.

10. Launay,G., Salza,R., Multedo,D., Thierry-Mieg,N. and Ricard-Blum,S. (2015) MatrixDB, the extracellular matrix interaction database: updated content, a new navigator and expanded functionalities. *Nucleic Acids Res.*, **43**, D321-327.

11. Licata,L., Briganti,L., Peluso,D., Perfetto,L., Iannuccelli,M., Galeota,E., Sacco,F., Palma,A., Nardozza,A.P., Santonico,E., *et al.* (2012) MINT, the molecular interaction database: 2012 update. *Nucleic Acids Res.*, **40**, D857-861.

12. Güldener,U., Münsterkötter,M., Oesterheld,M., Pagel,P., Ruepp,A., Mewes,H.-W. and Stümpflen,V. (2006) MPact: the MIPS protein interaction resource on yeast. *Nucleic Acids Res.*, **34**, D436-441.

13. Goll,J., Rajagopala,S.V., Shiau,S.C., Wu,H., Lamb,B.T. and Uetz,P. (2008) MPIDB: the microbial protein interaction database. *Bioinformatics*, **24**, 1743–1744.

14. Pagel,P., Kovac,S., Oesterheld,M., Brauner,B., Dunger-Kaltenbach,I., Frishman,G., Montrone,C., Mark,P., Stümpflen,V., Mewes,H.-W., *et al.* (2005) The MIPS mammalian protein-protein interaction database. *Bioinformatics*, **21**, 832–834.

15. Brown,K.R. and Jurisica,I. (2005) Online predicted human interaction database. *Bioinformatics*, **21**, 2076–2082.

16. Jiang,M., Ma,Y., Chen,C., Fu,X., Yang,S., Li,X., Yu,G., Mao,Y., Xie,Y. and Li,Y. (2009) Androgen-responsive gene database: integrated knowledge on androgen-responsive genes. *Mol. Endocrinol.*, **23**, 1927–1933.

17. Franceschini,A., Szklarczyk,D., Frankild,S., Kuhn,M., Simonovic,M., Roth,A., Lin,J., Minguez,P., Bork,P., von Mering,C., *et al.* (2013) STRING v9.1: protein-protein interaction networks, with increased coverage and integration. *Nucleic Acids Res.*, **41**, D808-815.

18. Szklarczyk,D., Morris,J.H., Cook,H., Kuhn,M., Wyder,S., Simonovic,M., Santos,A., Doncheva,N.T., Roth,A., Bork,P., *et al.* (2017) The STRING database in 2017: quality-controlled protein-protein association networks, made broadly accessible. *Nucleic Acids Res.*, **45**, D362–D368.

19. Blohm,P., Frishman,G., Smialowski,P., Goebels,F., Wachinger,B., Ruepp,A. and Frishman,D. (2014) Negatome 2.0: a database of non-interacting proteins derived by literature mining, manual annotation and protein structure analysis. *Nucleic Acids Res.*, **42**, D396-400.

20. Gene Ontology Consortium (2015) Gene Ontology Consortium: going forward. *Nucleic Acids Res.*, **43**, D1049-1056.

21. Kanehisa,M. and Goto,S. (2000) KEGG: Kyoto Encyclopedia of Genes and Genomes. *Nucl. Acids Res.*, **28**, 27–30.

22. Kanehisa,M., Sato,Y., Kawashima,M., Furumichi,M. and Tanabe,M. (2016) KEGG as a reference resource for gene and protein annotation. *Nucleic Acids Res.*, **44**, D457-462.

23. Kanehisa,M., Furumichi,M., Tanabe,M., Sato,Y. and Morishima,K. (2017) KEGG: new perspectives on genomes, pathways, diseases and drugs. *Nucleic Acids Res.*, **45**, D353–D361.

24. Schaefer,C.F., Anthony,K., Krupa,S., Buchoff,J., Day,M., Hannay,T. and Buetow,K.H. (2009) PID: the Pathway Interaction Database. *Nucleic Acids Res*, **37**, D674–D679.

25. Nishimura,D. (2001) BioCarta. *Biotech Software & Internet Report*, **2**, 117–120.

26. Croft,D., Mundo,A.F., Haw,R., Milacic,M., Weiser,J., Wu,G., Caudy,M., Garapati,P., Gillespie,M., Kamdar,M.R., *et al.* (2014) The Reactome pathway knowledgebase. *Nucl. Acids Res.*, **42**, D472–D477.

27. Fabregat,A., Sidiropoulos,K., Garapati,P., Gillespie,M., Hausmann,K., Haw,R., Jassal,B., Jupe,S., Korninger,F., McKay,S., *et al.* (2016) The Reactome pathway Knowledgebase. *Nucleic Acids Res.*, **44**, D481-487.

28. Kelder,T., van Iersel,M.P., Hanspers,K., Kutmon,M., Conklin,B.R., Evelo,C.T. and Pico,A.R. (2012) WikiPathways: building research communities on biological pathways. *Nucleic Acids Res.*, **40**, D1301-1307.

29. Kutmon,M., Riutta,A., Nunes,N., Hanspers,K., Willighagen,E.L., Bohler,A., Mélius,J., Waagmeester,A., Sinha,S.R., Miller,R., *et al.* (2016) WikiPathways: capturing the full diversity of pathway knowledge. *Nucleic Acids Res.*, **44**, D488-494.

30. Thomas,P., Durek,P., Solt,I., Klinger,B., Witzel,F., Schulthess,P., Mayer,Y., Tikk,D., Blüthgen,N. and Leser,U. (2015) Computer-assisted curation of a human regulatory core network from the biological literature. *Bioinformatics*, **31**, 1258–1266.
